# Age- and sex-related differences in social competence and emotion labeling in pre-adolescence

**DOI:** 10.1016/j.dcn.2024.101503

**Published:** 2024-12-24

**Authors:** Elizabeth E.L. Buimer, Pascal Pas, Carlijn van den Boomen, Mathijs Raemaekers, Rachel M. Brouwer, Hilleke E. Hulshoff Pol

**Affiliations:** a Department of Psychiatry, UMC Utrecht Brain Center, University Medical Center Utrecht, Utrecht University, Utrecht, the Netherlands; b Institute of Education and Child Studies, Leiden University, Leiden, the Netherlands; c Department of Experimental Psychology, Utrecht University, Utrecht, the Netherlands; d Department of Experimental Psychology, Helmholtz Institute, Utrecht University, Utrecht, the Netherlands; e Department of Neurology and Neurosurgery, UMC Utrecht Brain Center, University Medical Center Utrecht, Utrecht University, Utrecht, the Netherlands; f Department of Complex Trait Genetics, Centre for Neurogenomics and Cognitive Research, VU University, Amsterdam, the Netherlands

**Keywords:** Functional magnetic resonance imaging, Emotion processing, Social competence, Children, Development, Sex differences

## Abstract

Identification of facial expressions is important to navigate social interactions and associates with developmental outcomes. It is presumed that social competence, behavioral emotion labeling and neural emotional face processing are related, but this has rarely been studied. Here, we investigated these interrelations and their associations with age and sex, in the YOUth cohort (1055 children, 8–11 years old). Using a multistep linear modelling approach, we associated parent-reported social competence, basic emotion labeling skills based on pictures of facial expressions, and neural facial emotion processing during a passive-watching fMRI task with pictures of houses and emotional faces. Results showed better emotion labeling and higher social competence for girls compared to boys. Age was positively associated with emotion labeling skills and specific social competence subscales. These age- and sex-differences were not reflected in brain function. During fMRI, happy faces elicited more activity than neutral or fearful faces. However, we did not find evidence for the hypothesized links between social competence and behavioral emotion labeling, and with neural activity. To conclude, in pre-adolescents, social competence and emotion labeling varied with age and sex, while social competence, emotion labeling and neural processing of emotional faces were not associated with each other.

## Highlights

• Girls score higher than boys on social competence scales.
• Emotion labeling accuracy and speed are better in girls than in boys.
• Prosocial behavior and perspective taking are positively associated with age.
• Emotion labeling accuracy and speed are positively associated with age.
• No support for interrelations between social competence, emotion labeling and neural emotion processing in pre-adolescents.

## 1 Introduction

Social competence can be defined as the ability to engage in meaningful interactions with others (Junge et al., 2020). Emotion reasoning helps to navigate these social interactions. Emotion reasoning is the ability to make reasonably accurate inferences and predictions about the emotion states of other people (Ruba and Pollak, 2020). Emotion labeling is considered one of the building blocks of emotion reasoning (Ruba and Pollak, 2020). Emotion labeling is measured with verbal-response paradigms and thus requires language in contrast to other components of emotion reasoning, such as emotion categorization and emotion discrimination which can therefore be assessed earlier in life (Ruba and Pollak, 2020). In this manuscript we will use the term *emotion labeling* rather than the traditional term *emotion recognition* that is now considered less favorable (Barrett et al., 2019, Hoemann et al., 2020, Ruba and Pollak, 2020). Individuals differ in their ability to accurately label emotions on facial expressions and in the speed at which emotion labeling occurs. Accuracy and speed of emotion labeling are partly heritable (Swagerman et al., 2016) but also influenced by environmental factors such as childhood maltreatment (Assed et al., 2020, Bérubé et al., 2023).

Studies that investigated age and sex effects on social competence, suggested lower social competence in boys compared to girls (Maurice-Stam et al., 2018, Muris et al., 2003, Overgaauw et al., 2017) and increases in some aspects of social competence with age (Hawk et al., 2013, Marzocchi et al., 2002, Marzocchi et al., 2004). Emotion labelling skills also improve across development and may differ by sex. The developmental trajectories of emotion labelling are described in detail in Bayet and Nelson (2019). Due to the limited verbal abilities of young children, studies on early development focus on the differential processing of emotions rather than emotion labeling using either behavioral (e.g. habituation or preferential looking) or neurocognitive measurements (mostly electroencephalography (EEG)). Studies using such techniques showed that newborns do not show differential processing of facial emotional expressions, but this ability rapidly develops in the first year of life and continues to improve during childhood (Bayet and Nelson, 2019). Studies in school-age children investigated the development of emotion labelling. In general, older children are faster and more accurate in emotion labeling (Herba and Phillips, 2004), but age effects on emotion labeling accuracy and reaction time continue to exist throughout pre-adolescence (Gur et al., 2012, Verpaalen et al., 2019). Looking at specific emotional expressions, the ability to accurately label happy faces is thought to develop first (Riddell et al., 2024). Around the age of 5, most children can accurately label happy faces (Durand et al., 2007), while the identification of fearful emotions is thought to have a more protracted developmental trajectory (Bayet and Nelson, 2019, Durand et al., 2007). Regarding sex effects, a meta-analysis reports a female advantage in emotion labeling skills (McClure, 2000). The effect of sex is small and relatively constant throughout development.

Age- and sex-related variation in emotion labeling skills and social competence may also reflect variation in neurocognitive differentiation of emotional expressions. Most studies using EEG show no effects of age on differential responses to emotional stimuli in late childhood and early adolescence (see Dickey et al., 2021 for a review), although many but not all studies show general developmental changes in brain responses to emotional faces (Dickey et al., 2021, Bigelow et al., 2021; Ramos-Loyo et al., 2024). In addition to EEG, functional MRI (fMRI) can be used to study the neural processing of emotional faces. There are only a few studies available on neural correlates of emotion processing in pre-adolescents. One recent large study in 759 children, adolescents and adults (ages 8–23 years) showed a developmentally stable modular architecture with the strongest developmental changes in frontoparietal circuits (Zhang et al., 2019). Relatively stable patterns of activation across development were also found in a study of 823 children between 5- and 15-years-old (Camacho et al., 2023).

The neural basis of facial-emotion processing requires different levels of specialization, as faces convey a range of hierarchically embedded information (Adolphs, 2002, Bayet and Nelson, 2019). Developmental studies suggest that separate processes underlie the perception of emotional faces and the processing of other facial information such as identity, even though these processes can affect each other (Bayet and Nelson, 2019). A meta-analysis on children and adults (mean age 27 years; 105 fMRI studies) on the processing of emotional faces showed that emotional faces elicited activity in several visual, limbic, temporoparietal and prefrontal areas; the putamen; and the cerebellum (Fusar-Poli et al., 2009). Neural activity in the visual cortex and cerebellum was observed independent of emotional valence. Happy, fearful, and sad faces specifically activated the amygdala. Disgusted and angry faces specifically activated the insula. A recent meta-analysis in adults (141 fMRI and PET studies) showed consistent activity in the left amygdala in response to happy, angry, fearful and sad faces, but category-specific lateralization of the ventromedial prefrontal cortex (Xu et al., 2021). Another recent meta-analysis in adults (96 fMRI and PET studies) showed that the ventral pathway, especially the left fusiform gyrus, was more responsive to facial expression than the dorsal pathway (Liu et al., 2021).

So far, behavioral emotion labeling, neural processing of emotional faces and social competence are mostly studied in isolation. Still, previous studies show that both lower emotion labeling accuracy and atypical neural processing of emotional faces relate to neurodevelopmental conditions often associated with atypical social competence. For instance, a meta-analysis showed that more accurate emotion labeling is associated with higher social competence and less behavioral problems in childhood and adolescence (Trentacosta and Fine, 2010). Moreover, reviews and meta-analyses suggest that lower emotion labeling accuracy may be associated with a wide variety of neuropsychiatric or neurodevelopmental conditions, such as autism spectrum disorder (Harms et al., 2010, Uljarevic and Hamilton, 2013, Yeung, 2022), mood disorders, anxiety disorders or attention deficit hyperactivity disorder (Collin et al., 2013), internalizing problems (Zhang et al., 2024) and externalizing problems (Cooper et al., 2020). Furthermore, aberrant neural processing of emotional faces is one of the most consistent neuroimaging findings in the childhood maltreatment literature (Hein and Monk, 2017) and is related to various psychiatric conditions (Delvecchio et al., 2013, Etkin and Wager, 2007, Harms et al., 2010, Mitchell et al., 2014, Stuhrmann et al., 2011, Monteiro et al., 2017). However, to our current knowledge no studies investigated both emotional labeling accuracy and neural emotional face processing in relation to social competence in a large population-based developmental cohort. Investigating interrelations between all three aspects may help further the understanding of mechanisms underlying social behavior and (a)typical development.

In this study we aim to investigate age- and sex-effects on social competence, behavioral emotion labeling and neural processing of facial expressions of emotions in pre-adolescence. Furthermore, we are interested in the link between inter-individual differences in emotion labeling accuracy and reaction time, neural facial-emotion processing, and social competence. We hypothesize 1) that older children and girls are faster and more accurate when labeling emotions and score higher on all social competence subscales; 2) that shorter emotion labeling reaction time and higher accuracy is related to higher social competence; 3) that variation in neural processing of emotional faces can be partly explained by age, sex and emotion labeling skills, with older children, girls and children with superior emotion labeling skills showing different activation patterns; 4) that social competence correlates with brain activation patterns in response to emotional versus neutral faces.

## 2 Materials and methods

### 2.1 Participants

**Fig. 1:**
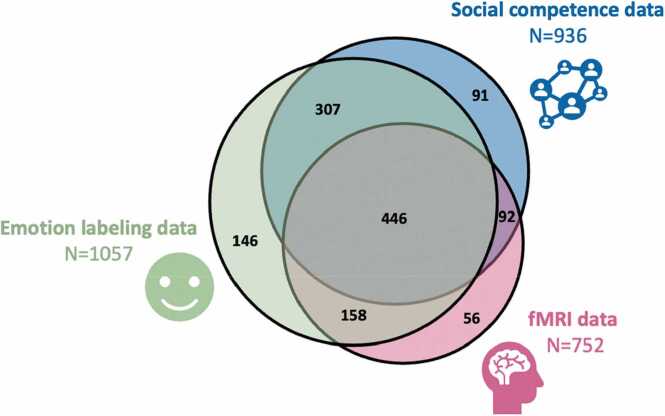
Venn diagram of the available data. Labels specify the data domains of interest for this study and the total number of participants with data for the domain. Colors of the labels correspond to the colors of the three circles. Area of the circles and the overlapping spheres are proportional, and numbers indicate absolute numbers of children. Figure adapted from web application DeepVenn (Hulsen, 2022).

The YOUth cohort study is a longitudinal population-based study on brain development with a specific focus on social competence and self-regulation (Onland-Moret et al., 2020). In the current study we included data from the first wave of YOUth: Child & Adolescent, in which 1332 children between 7.9 and 11.0 years old participated (57 % female). In the YOUth study data on sex and gender identity is collected, but for this study we focused on sex-effects. The YOUth cohort study was conducted in Utrecht, a province of the Netherlands, with on average highly educated inhabitants with high incomes (Fakkel et al., 2020, Buimer et al., 2022). All data included here was collected prior to the COVID-19 pandemic. This study was approved by the Medical Research Ethics Committee Utrecht. Children participated on a voluntary basis and parents/guardians gave written consent and assent. Fig. 1 shows the available data for the domains relevant to the current study.

### 2.2 Social competence data

Social competence was defined using the subscales *perspective taking* and *empathic concern* of the Interpersonal Reactivity Index (IRI, self-report, Davis, 1983) and the subscales *prosocial behaviour* and *peer problems* of the Strengths and Difficulties Questionnaire (SDQ, parent-report, Goodman, 1997, Goodman, 2001). Together the subscales of the two questionnaires tap different aspects of social competence (Junge et al., 2020). Each of the four subscales contains 5 items, which were summed to get total subscale scores for each child.

### 2.3 Behavioral emotion labeling

The Penn Computerized Neurobehavioral Battery (CNB) is developed by the University of Pennsylvania to capture specific cognitive domains that link to brain function (Gur et al., 2010). Within YOUth: Child & Adolescent, a subset of the web-based CNB was collected, including the 40-item Emotion Recognition Test (ER-40). In the ER-40, the child labels the emotion on presented images of facial expressions in a multiple-choice format: happy, sad, anger, fear or neutral. The multiple-choice options were presented in the children’s native language, Dutch. From the ER-40, we used accuracy (the number of correct responses) and reaction time (the median response times computed over the trials with correct identifications). We did not observe irregularities in the data due to non-compliance (for example, continuously picking the same answer). One participant had a response time for fearful facial expressions of 11.6 seconds. The participant with this extreme outlier was removed from the dataset because the median response time for fearful faces was based on only this one correct trial resulting in a response time 13 standard deviations from the mean of 2.5 seconds. Other outliers were not as extreme or based on more than one trial. As we were interested in inter-individual variation, we did not remove these other outliers. Boxplots of reaction times in relation to correct responses with and without the outlier can be found in Supplementary figure S1.

### 2.4 Neuroimaging data

#### 2.4.1 Stimuli presentation

**Fig. 2:**
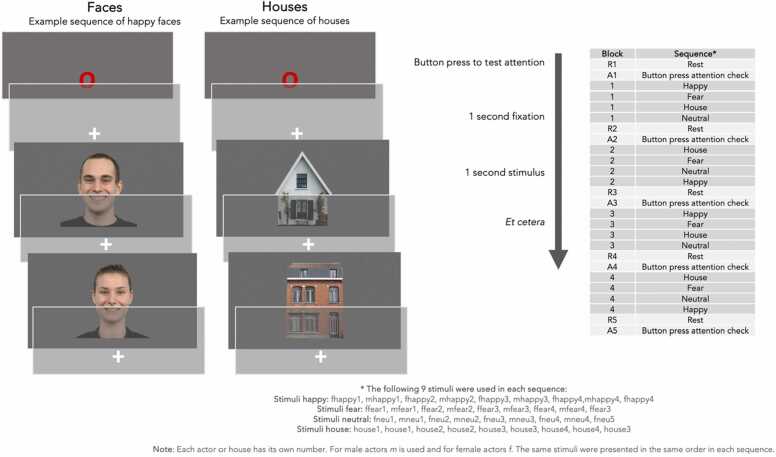
Design of fMRI task.

The face/house fMRI task is a passive viewing task in which children are presented with four blocks of stimuli (Fig. 2). Each block contains 4 sequences of 9 stimuli. The sequences contain pictures of one of the following categories: pictures of faces with a specific emotional valence (happy, fearful or neutral expression) or pictures of houses. The same pictures are used in each block with for each sequence 9 different pictures of the same category in a row. Within each block, categories (sequences) appear in a semi-random order. Stimuli are presented in blocks of 18 seconds. Within each block, stimuli are presented for 1 second followed by a 1 second fixation cross. Between blocks there is a period of rest. To ensure that the children remain focused, they are instructed to press a button when a red circle appears in the center of the screen. This circle appears as first stimulus at the start of the task and after each block (five times in total). No other behavioral data is collected during the scan. For the pictures of the faces, we used different stimuli than in the behavioral emotion labeling task. In the fMRI task we used stimuli from the Radboud Faces Database from 9 adult actors (4 male, 5 female) (Langner et al., 2010). The stimuli were presented on an MRI-compatible 23-inch LCD screen with a resolution of 1080 by 1920 pixels (BOLDscreen, Cambridge Research Systems).

#### 2.4.2 Neuroimaging acquisition

In the YOUth cohort study, the collection of MRI data is monitored closely over time based on human data and weekly collected phantom data. The YOUth MRI protocol, quality control and test-retest reliability are described in detail elsewhere (Buimer et al., 2020). All data was acquired on the same Philips Ingenia CX 3.0 T MRI scanner. Whole-brain, T2 * -weighted echo planar images were acquired with the following parameters: TR = 1000 ms; TE = 25 ms; flip angle 65°; 2.5 mm × 2.5 mm in-plane resolution; 2.5 mm slice thickness; 51 slices per volume; SENSE factor 1.8 (anterior–posterior); multiband factor 3. Data was acquired in a single run of 389 dynamic scans. For anatomical reference a structural T1-weighted 3D gradient echo scan was acquired with the following parameters: TR = 10 ms; TE = 4.6 ms; flip angle = 8°; voxel size = 0.75 mm× 0.75 mm× 0.80 mm; parallel imaging factor = 1.70 (AP) and 1.40 (RL).

#### 2.4.3 Preprocessing

Preprocessing and subsequent processing of fMRI scans were done using SPM12 (http://www.fil.ion.ucl.ac.uk/spm/) in MATLAB 2020b (The MathWorks Inc., Massachusetts, United States). The steps described here are identical to those used in previous studies that included YOUth fMRI data (Buimer et al., 2020, Pas et al., 2021). In short, preprocessing included realignment to correct for head motion, where the time-series were registered by a least-square approach and a rigid-body transformation. After realignment, slice timing correction, spatial normalization to MNI-152 space, and smoothing (8 mm full width at half maximum) to correct for inter-individual differences in functional anatomy were applied. Collected MRI-scans of the children are processed immediately after data collection for quality control purposes, on a local server with scripted pipelines. For each scan a quality control report was generated (Buimer et al., 2022). Reports contained figures of realignment parameters, motion statistics and signal measures plotted against the time series. Furthermore, signal and noise brain maps were included in the reports. Local drops in signal were found in scans with high framewise displacement scores but signal drops could also indicate scanner artefacts. We found that the best and most objective way to remove scans with severe motion artefacts was by using a fixed fMRI signal threshold (see individual analyses).

#### 2.4.4 Individual analyses

Task activity was estimated using a general linear model (GLM) including factors for happy faces, fearful faces, neutral faces, and houses. The six realignment parameters were added to the design matrix to model residual effect of head motion. All data were high-pass filtered with a cut-off of 128 seconds to remove low-frequency drifts. We used a global signal threshold of 80 % to avoid including brain areas with low signal. Participants exhibiting significant signal drops within the brain mask, leading to holes in the mask, were excluded from the analysis (Pas et al., 2021). This resulted in exclusion of 53 out of 806 fMRI scans. Low signals drops were mostly related to motion artefacts, although in some cases scanner artefacts may have played a role. After the GLM, we defined four contrasts: 1) faces > houses; 2) happy faces > neutral faces; 3) fearful faces > neutral faces; 4) fearful faces > happy faces. The first-level analyses produced four contrast maps for each participant.

#### 2.4.5 Group analyses

**Fig. 3:**
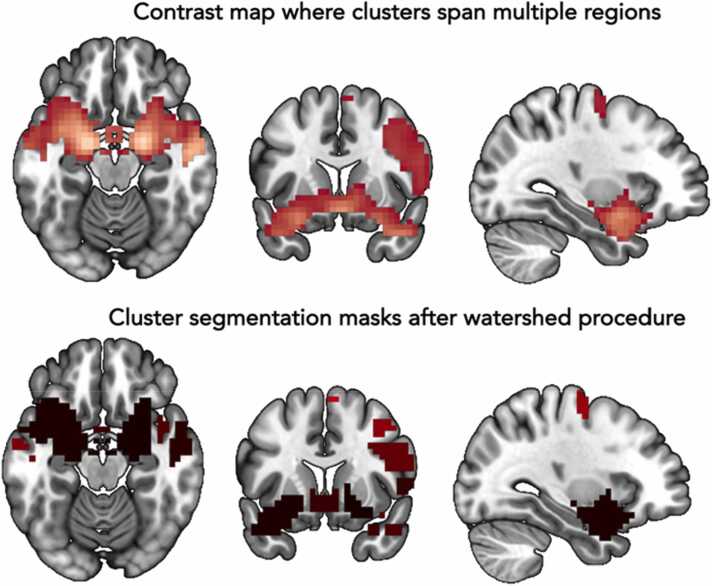
Example of the watershed procedure. As input, we use a contrast map (in this example Faces > Houses) thresholded with *p*_FWE_ < .05 and a cluster extent threshold based on *p* < .001 which corresponds to a z-value of 3.1. The watershed procedure then finds local peaks and minima and creates binary masks of the segmentations. The segmentation masks can be used as regions-of-interest in subsequent analyses.

In the second-level analyses, task activation maps were thresholded at *p*_*FWE*_ < .05 and a cluster extent threshold based on *p* < .001 which corresponds to a *z*-value of 3.1 (based on Eklund et al., 2016). The threshold for significance was converted into a voxel size threshold (*k*) based on the SPM file of each contrast using the SPM Cluster Size Threshold Estimation tool (https://doi.org/10.5281/zenodo.1689891). This resulted in a cluster size threshold of 21 voxels for contrast 1 (faces > houses); 27 voxels for contrast 2 and 4 (happy faces > neutral faces and fearful faces > happy faces); 28 voxels for contrast 3 (fearful faces > neutral faces). Because we found widespread and very large clusters (even with our stringent thresholds), we included a watershed procedure to subdivide clusters based on local minima and maxima. The peaks and local minima were used to define borders and to split the cluster into separate segments (Fig. 3). Individual contrast maps were masked with the different segments and the average of the *β-*values for the voxels within a mask were extracted for subsequent analyses.

### 2.5 Statistical analyses

All statistical analyses were conducted in R version 4.0.5 (2021-03-31). The effect of age and sex on social competence was tested with separate linear models for each social competence subscale. The threshold for significance was adjusted to *p* < .0125 based on Bonferroni correction for the four subscales.

For the emotion labeling data, we started with an analysis of variance (ANOVA) to test if the median response time on correct trials was on average different for different types of emotions. Tukey's test was used as post-hoc analysis to test for differences in group means. Next, we investigated the effect of age and sex on emotion labeling (accuracy and response time) using separate linear models for the different emotions. The threshold for significance was adjusted to *p* < .005 based on a Bonferroni correction for the 10 analyses (5 contrasts for both accuracy and speed). Additionally, we reran these analyses after adding the 4 social competence subscales using the same Bonferroni corrected threshold for significance.

For the fMRI analyses, the average *β*-values in individual segments were the dependent variables in linear regression models. As independent variables we started off with only age and sex. Next, we added emotion labeling skills: response time and accuracy for happy emotions as predictors for active subclusters in the happy versus neutral contrast; response time and accuracy for fearful emotions as predictors for active subclusters in the fearful versus neutral contrast; response time and accuracy independent of emotional valence as predictors for the faces versus houses contrast. We controlled the number of false positives by adjusting the *p*-values over the different subclusters for the false discovery rate (*FDR*) within each contrast and using a threshold of *p*_*FDR*_ < .05 (Benjamini and Hochberg, 1995). Lastly, we ran these analyses again with the four social competence subscales as predictors in addition to age and sex, instead of emotion labeling skills, again using *FDR*-adjusted *p-*values for determining significance.

### 2.6 Addressing non-normality with residual-based permutations

In the case when dependent variables were not normally distributed, we ran the linear models as usual to get effect sizes, standard errors and the t-statistic of the variable of interest but determined significance by computing *p*-values through residual-based permutations (Buzkova, 2016). For each variable of interest, a separate model was fitted leaving this variable out of the equation, which acted as the null model in this analysis. Next, residuals of this null model were used to create new observations with the same sample size as the original sample. First, we computed the fitted values for each observation and added permuted residuals. The effect of the variable of interest (left out in the null-model) was tested in the permuted sample by fitting the full linear model including the variable of interest, providing a t-statistic for this variable. This procedure was repeated 10.000 times resulting in a distribution of *t*_i_-statistics for the variable of interest. Finally, the *p*-value was calculated by assessing the probability of the *t*-statistic of the original model (*t*_orig_) given the *t*_i_ distribution:

*p*_permutated_= (1+sum(abs(*t*_i_)> =abs(*t*_orig_)))/(10000+1)

This procedure is repeated for each variable of interest. Illustrative examples of this procedure can be found in Figure S2.

### 2.7 Post-hoc analyses

#### 2.7.1 ROI analyses

After the data were seen and after preprinting our study (Buimer et al., 2024), we added region of interest (ROI) analyses. ROIs were selected for each contrast separately (Table 2) based on literature (Fusar-Poli et al., 2009, Xu et al., 2021, Liu et al., 2021, Passarotti et al., 2003). Cerebellar ROIs were not included as a small field-of-view was used during the fMRI acquisition and the prefrontal cortex was prioritized over the cerebellum while setting the field-of-view. The automated anatomical labelling (AAL) template (Tzourio-Mazoyer et al., 2002) was used to generate mean activation levels per AAL region. A one-sample *t*-test was used to test if the group mean of task activation in a ROI differed from zero. ROIs with an *FDR*-adjusted *p*-value < .05 were used in subsequent analyses. Next, the mean activation level in individual ROIs were the dependent variables in linear regression models. Similar to described in Section 2.5, we started with the effect of age and sex, then emotion labeling skills and then social competence subscales on activation in the ROIs. Again, within each contrast *p*-values were *FDR*-adjusted over the different ROIs to determine significance.

**Table 1:** The effect of age and sex on emotion labeling accuracy and speed. The results of linear models with age and sex as independent variables and number of correct responses or median reaction time in milliseconds based on correct trials only as dependent variables. Each row shows the results of a separate linear model for a specific emotion. The subscript orig indicates that the statistics are computed from the original linear model (*ß*_orig_, SE_orig_, *t*_orig_) while the subscript permutated indicates that the *p*-values are computed from the residual-based permutations (*p*_permutated_).

	Model	Age				Sex			
	Df	*ß*_orig_	SE_orig_	*t*_orig_	*p*_permutated_	*ß*_orig_	SE_orig_	*t*_orig_	*p*_permutated_
Accuracy									
Happy	1054	0.05	0.02	2.05	.0404	0.12	0.04	3.06	.0023*
Sad	1054	0.18	0.06	3.23	.001*	0.37	0.10	3.80	<.0001*
Angry	1054	0.28	0.05	5.64	<.001*	0.15	0.09	1.73	.0839
Fearful	1054	0.29	0.06	5.12	<.001*	0.32	0.10	3.33	.0011*
Neutral	1054	0.23	0.06	3.98	<.001*	−0.10	0.10	−1.01	.3219
Reaction time									
Happy	1054	−150	14	−10.77	<.0001*	−96	24	−4.00	.0002*
Sad	1050	−145	26	−5.62	<.0001*	−97	45	−2.17	.0296
Angry	1051	−181	28	−6.41	<.0001*	−156	49	−3.20	.0022*
Fearful	1054	−213	28	−7.51	<.0001*	−67	49	−1.37	.1684
Neutral	1043	−253	31	−8.11	<.0001*	18	54	0.33	.7465

* = survives Bonferroni correction for the total number of analyses in the table, i.e., *p* < .005.

**Table 2:** Task activations in regions-of-interest. For comparison ROI-based analyses with and without smoothing as part of the preprocessing pipeline are included. ROIs were created using the automated anatomical labelling (AAL) template (Tzourio-Mazoyer et al., 2002).

	**Original group**				**Low motion subgroup**			
	**Smoothed data**		**Non-smoothed data**		**Smoothed data**		**Non-smoothed data**	
**Contast and AAL Region**	***t*****(752)**	***p***_***FDR***_	***t*****(752)**	***p***_***FDR***_	***t*****(717)**	***p***_***FDR***_	***t*****(717)**	***p***_***FDR***_
Faces > Houses								
Fusiform_L	−39.353	<0.0001*	−39.748	<0.0001*	−38.554	<0.0001*	−38.854	<0.0001*
Fusiform_R	−43.511	<0.0001*	−47.229	<0.0001*	−42.554	<0.0001*	−46.020	<0.0001*
Occipital_Sup_L	−38.33	<0.0001*	−38.020	<0.0001*	−37.791	<0.0001*	−37.316	<0.0001*
Occipital_Sup_R	−36.793	<0.0001*	−36.596	<0.0001*	−35.988	<0.0001*	−35.643	<0.0001*
Occipital_Mid_L	−43.674	<0.0001*	−44.38	<0.0001*	−43.158	<0.0001*	−43.452	<0.0001*
Occipital_Mid_R	−42.576	<0.0001*	−44.243	<0.0001*	−42.020	<0.0001*	−43.572	<0.0001*
Occipital_Inf_L	−23.52	<0.0001*	−22.281	<0.0001*	−23.445	<0.0001*	−22.262	<0.0001*
Occipital_Inf_R	−32.818	<0.0001*	−31.378	<0.0001*	−32.560	<0.0001*	−31.008	<0.0001*
Parietal_Sup_L	−12.691	<0.0001*	−13.868	<0.0001*	−12.166	<0.0001*	−13.215	<0.0001*
Parietal_Sup_R	−10.243	<0.0001*	−11.557	<0.0001*	−9.940	<0.0001*	−11.130	<0.0001*
Parietal_Inf_L	−1.984	0.0556	−2.867	0.0046*	−1.758	0.0924	−2.615	0.0098*
Parietal_Inf_R	−1.871	0.0664	−3.080	0.0025*	−1.608	0.1166	−2.778	0.0065*
Temporal_Inf_L	0.440	0.6597	−0.447	0.655	0.782	0.4346	−0.049	0.9609
Temporal_Inf_R	−3.312	0.0012*	−4.183	<0.0001*	−2.961	0.0040*	−3.739	0.0003*
Happy > Neutral								
Fusiform_L	13.178	<0.0001*	12.699	<0.0001*	13.120	<0.0001*	12.459	<0.0001*
Fusiform_R	12.467	<0.0001*	12.359	<0.0001*	12.238	<0.0001*	11.893	<0.0001*
Amygdala_L	6.219	<0.0001*	6.647	<0.0001*	6.579	<0.0001*	6.906	<0.0001*
Amygdala_R	6.613	<0.0001*	6.590	<0.0001*	6.891	<0.0001*	6.679	<0.0001*
Cingulum_Ant_L	5.255	<0.0001*	6.097	<0.0001*	4.782	<0.0001*	5.661	<0.0001*
Cingulum_Ant_R	4.535	<0.0001*	4.446	<0.0001*	3.918	0.0001*	3.839	0.0002*
Occipital_Mid_L	18.657	<0.0001*	18.934	<0.0001*	19.010	<0.0001*	19.322	<0.0001*
Occipital_Mid_R	14.331	<0.0001*	14.503	<0.0001*	14.225	<0.0001*	14.353	<0.0001*
Precuneus_L	5.774	<0.0001*	5.343	<0.0001*	5.900	<0.0001*	5.464	<0.0001*
Precuneus_R	5.494	<0.0001*	5.017	<0.0001*	5.659	<0.0001*	5.136	<0.0001*
Insula_L	3.628	0.0003*	3.408	0.0008*	3.346	0.0010*	3.074	0.0024*
Insula_R	5.098	<0.0001*	5.491	<0.0001*	4.794	<0.0001*	5.223	<0.0001*
Frontal_Med_Orb_L	2.680	0.0079*	1.504	0.1331	2.999	0.0029*	1.920	0.0552
Frontal_Med_Orb_R	2.356	0.0187*	1.566	0.1241	2.570	0.0104*	1.965	0.0524
Putamen_L	5.145	<0.0001*	4.798	<0.0001*	4.973	<0.0001*	4.639	<0.0001*
Putamen_R	5.119	<0.0001*	5.020	<0.0001*	4.809	<0.0001*	4.765	<0.0001*
SupraMarginal_L	5.387	<0.0001*	5.276	<0.0001*	5.632	<0.0001*	5.468	<0.0001*
SupraMarginal_R	5.200	<0.0001*	5.345	<0.0001*	5.305	<0.0001*	5.449	<0.0001*
Temporal_Mid_L	7.299	<0.0001*	7.355	<0.0001*	7.591	<0.0001*	7.389	<0.0001*
Temporal_Mid_R	5.926	<0.0001*	5.759	<0.0001*	5.970	<0.0001*	5.619	<0.0001*
Fearful > Neutral								
Fusiform_L	7.832	<0.0001*	8.701	<0.0001*	7.828	<0.0001*	8.547	<0.0001*
Fusiform_R	6.737	<0.0001*	7.354	<0.0001*	6.443	<0.0001*	6.923	<0.0001*
Amygdala_L	6.067	<0.0001*	6.710	<0.0001*	7.361	<0.0001*	7.825	<0.0001*
Amygdala_R	5.545	<0.0001*	5.457	<0.0001*	5.895	<0.0001*	5.569	<0.0001*
Parietal_Inf_L	−1.153	0.448	−1.261	0.3322	−1.231	0.4372	−1.329	0.3684
Parietal_Inf_R	−0.037	0.9705	−0.012	0.9901	−0.362	0.7450	−0.390	0.7255
Frontal_Med_Orb_L	−1.081	0.448	−1.806	0.1606	−0.870	0.5769	−1.506	0.2983
Frontal_Med_Orb_R	−0.710	0.5735	−1.003	0.4065	−0.496	0.7317	−0.595	0.6516
Frontal_Inf_Oper_L	1.040	0.448	1.224	0.3322	0.875	0.5769	0.965	0.4724
Frontal_Inf_Oper_R	0.741	0.5735	0.751	0.5434	0.325	0.7450	0.351	0.7255
Frontal_Inf_Tri_L	1.392	0.3286	1.468	0.285	1.098	0.4909	1.111	0.4367
Frontal_Inf_Tri_R	0.947	0.476	1.037	0.4065	0.453	0.7317	0.555	0.6516
Frontal_Inf_Orb_L	1.961	0.1131	1.281	0.3322	1.705	0.1994	1.128	0.4367
Frontal_Inf_Orb_R	2.117	0.089	1.952	0.1321	1.805	0.1840	1.588	0.2898
Occipital_Inf_L	11.413	<0.0001*	11.887	<0.0001*	11.500	<0.0001*	12.030	<0.0001*
Occipital_Inf_R	13.479	<0.0001*	13.428	<0.0001*	13.771	<0.0001*	13.700	<0.0001*
Pallidum_L	−0.097	0.9705	−0.608	0.5977	−0.466	0.7317	−0.952	0.4724
Pallidum_R	−0.437	0.7451	−0.576	0.5977	−0.552	0.7317	−0.576	0.6516
Fearful > Happy								
Amygdala_L	−0.558	0.5768	−0.502	0.6155	0.159	0.8736	0.226	0.8212
Amygdala_R	−1.292	0.2624	−1.505	0.177	−1.221	0.2965	−1.506	0.1766
Cingulum_Ant_L	−8.503	<0.0001*	−9.184	<0.0001*	−8.187	<0.0001*	−8.918	<0.0001*
Cingulum_Ant_R	−7.267	<0.0001*	−7.154	<0.0001*	−6.894	<0.0001*	−6.907	<0.0001*

* = Survives a threshold < 0.05 after adjusting for the False Discovery Rate (FDR).

#### 2.7.2 The effects of smoothing

Even though a smoothing kernel of 8 mm is still widely used (Xu et al., 2021), for large developmental cohort studies this level of smoothing may be too much (Gardumi et al., 2016, Jo et al., 2007; Sacchet and Knutson, 2013). To assess the impact of smoothing on our data, ROI-analyses were run with and without smoothing. All above-described analyses were run twice to be able to compare smoothed and non-smoothed output. When reporting the whole brain task effects based on the non-smoothed data, we used the same thresholding as for the smoothed data: *p*_*FWE*_ < .05 and a cluster extent threshold based on *p* < .001 which corresponds to a *z*-value of 3.1 (based on Eklund et al., 2016). For the non-smoothed data this resulted in a cluster extent threshold of k = 2 for all contrasts.

#### 2.7.3 The effects of motion artefacts

To investigate potential effects of motion contamination in our data, we added a frame-to-frame metric for exclusion. We created a low motion subgroup of children by excluding children with high motion frames (> 0.3 mm, based on Smith et al., 2022) on over 30 % of the frames, thus retaining only children with at least 70 % low motion frames (n = 718). ROI-based analyses were rerun for this low motion subgroup with and without smoothing.

## 3 Results

### 3.1 Social competence

#### 3.1.1 Variation

For the Strengths and Difficulties Questionnaire (SDQ) scores ranged from 1 to 10 for *prosocial behavior* and 0–9 for *peer problems* (theoretical range for both subscales is from 0 to 10). As can be expected in a cohort study, the SDQ subscales were skewed towards typical socio-emotional behavior with a mean and standard deviation of 8.49 (1.69) for *prosocial behavior* and 1.07 (1.56) for *peer problems*. For the Interpersonal Reactivity Index (IRI) both subscales (*perspective taking* and *emphatic concern*) ranged from 0 to 28 covering the full range of possible scores. The data was normally distributed with a mean and standard deviation of 14.37 (4.96) for *perspective taking* and 18.44 (4.40) for *emphatic concern*.

#### 3.1.2 The effects of age and sex

**Fig. 4:**
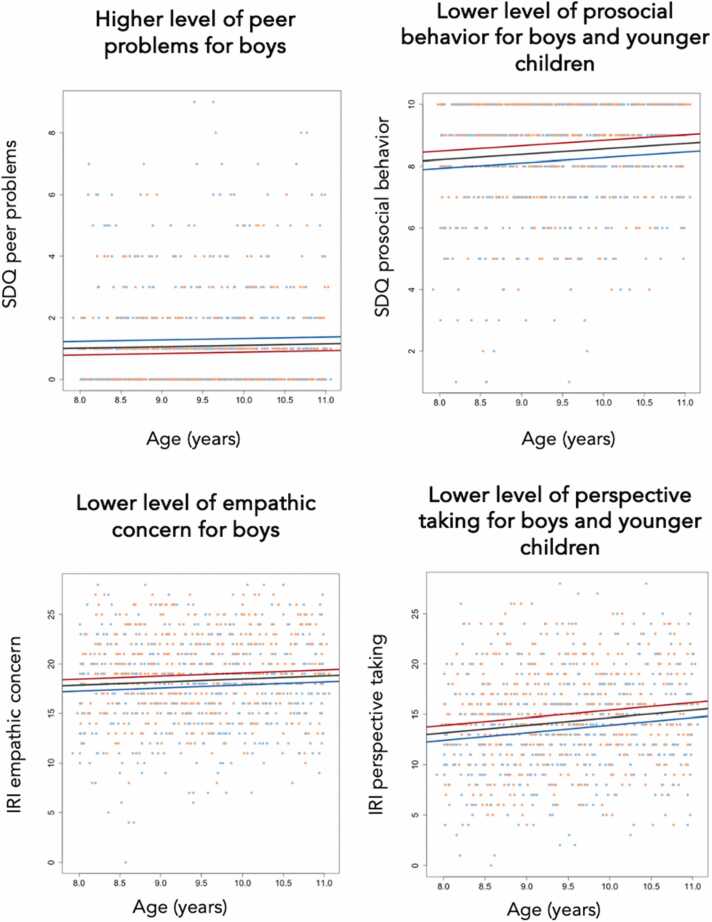
Effects of age and sex on social competence subscales. Red dots indicate social competence scores for girls and blue dots indicate scores for boys. Lines show the relation modeled linearly between social competence score and age (for girls in red, boys in blue and in black for the group as whole). Peer problems and prosocial behavior are subscales from the for the Strengths and Difficulties Questionnaire (SDQ). Empathic concern and perspective taking are subscales from the Interpersonal Reactivity Index (IRI).

Better social competence was found for girls compared to boys for all subscales and age effects were found for two subscales (Fig. 4). From the SDQ, *prosocial behavior* increased with age *t*(933) = 2.731, *β* = 0.175, standard error = 0.064, *p*_*permutated*_ = .0066 and was higher for girls *t*(933) = 5.096, *β* = 0.557, standard error *=* 0.109*, p*_*permutated*_ = .0001. No age effects were found for *peer problems* and higher scores were reported for boys compared to girls *t*(933) = -4.314, *β* = -0.443, standard error *=* 0.103*, p*_*permutated*_ = .0002. From the IRI, *perspective taking* increased with age *t*(933) = 3.983, *β* = 0.751, standard error = 0.188, *p*_*permutated*_ = .0001 and was higher for girls *t*(933) = 4.645, *β* = 1.488, standard error *=* 0.320*, p*_*permutated*_ = .0001. No age effects were found for *empathic concern* and lower scores were reported for boys compared to girls *t*(933) = 4.252, *β* = 1.211, standard error *=* 0.285*, p*_*permutated*_ = .0001. Reported results survived Bonferroni correction based on the four subscales (*p* < .0125).

### 3.2 Emotion labeling skills

#### 3.2.1 Accuracy and response time

**Fig. 5:**
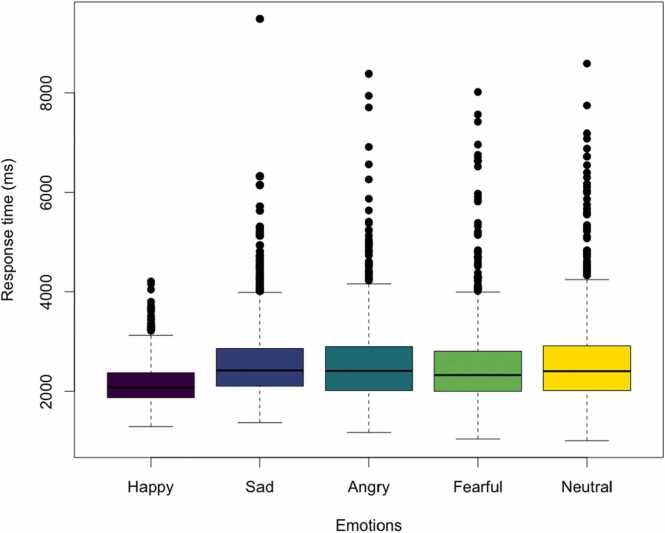
Response time on correct trials for the different emotions. Boxplot of the quartiles of the median response times on correct trials for different facial expressions of emotion. Each color represents a different facial expression stimuli. Black dots are individual data points outside the interquartile range.

Children were highly accurate when labeling happy, fearful, and neutral facial expressions in all trials, while some angry and sad faces proved more difficult on average (Figure S3). The mean and standard deviations of the accuracy measures were: Happy 7.64 (0.62), Sad 4.89 (1.56), Angry 4.28 (1.40), Fearful 6.53 (1.59), Neutral 6.92 (1.62). The difference in accuracy between emotions was significant *F*(4)= 1067, *p* < .001. The Tukey post-hoc test revealed that all cross comparisons showed significant differences. There was a significant difference in median response time on correct trials between the different emotions, *F*(4)= 66.93, *p* < .001. The Tukey post-hoc test revealed that children were faster on correct trials for happy faces compared to each of the other emotions (all *p* < .001), with no statistical differences between the other emotions (Fig. 5). When repeating the analysis limiting to children with at least 4 correct responses on every type of emotion (n = 258), we found the same results. The relation between the median response time on correct trials and the number of correct trials for each child can be found in Supplementary figure S1.

#### 3.2.2 The effects of age and sex

As the distribution of the emotion labeling data violated assumptions of normality, we tested for significance with residuals-based permutations (Buzkova, 2016). For more information on this procedure, see the supplementary materials and Figure S2. In general, older children and girls had an advantage and were significantly more accurate and faster on accurate trials when labeling most of the emotions (Table 1). The accuracy when labeling sad, angry, fearful, and neutral emotions was significantly higher in older children. For happy emotions, the effect of age on accuracy did not survive Bonferroni correction (*p* = .0404). There was a statistically significant advantage for older children in the reaction time for correctly labeling all emotions. Furthermore, girls were more accurate when labeling happy, sad, and fearful facial expressions, and faster in the correct trials for happy and angry facial expressions. See supplementary table S1 for all the statistics.

#### 3.2.3 The relation with social competence

Again, residuals-based permutations were used to determine significance (Buzkova, 2016). See supplementary methods and Figure S2. None of the four subscales of social competence were significant predictors of emotion labeling accuracy or speed for any of the emotions in linear models corrected for age and sex (see Supplementary table S2).

### 3.3 The neural processing of emotional faces

#### 3.3.1 Task compliance

Task compliance was good with most children responding to every red circle in between blocks of the stimuli of interest (Figure S4). Only 2.3 % of the children (n = 18) showed low task compliance, which was defined as pushing the button in between blocks never (n = 7), only once (n = 8) or twice (n = 3). Because the percentage of children with low task compliance was so low, all children were included in subsequent analyses.

#### 3.3.2 Task activation

**Fig. 6:**
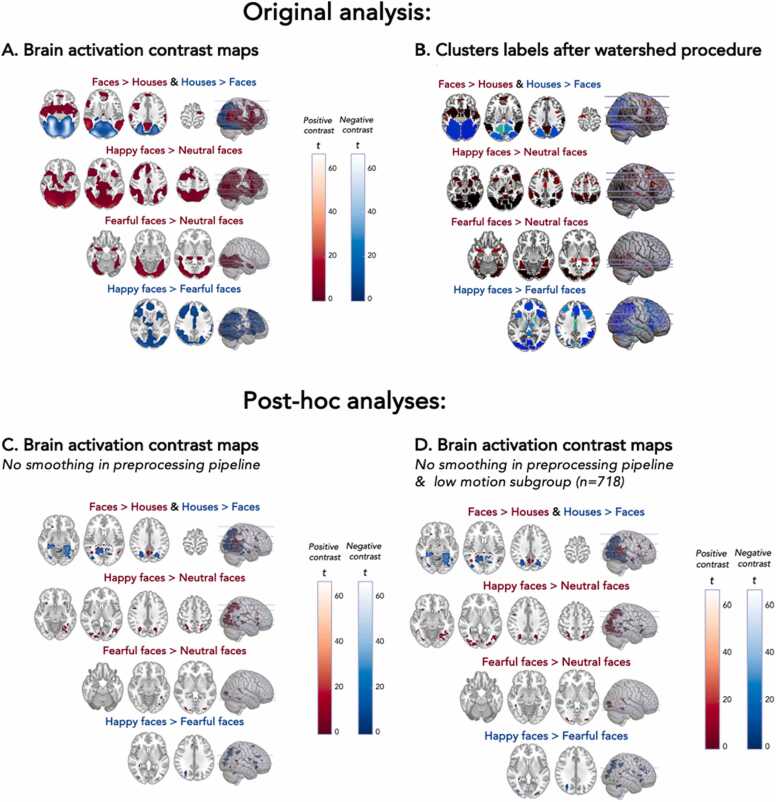
From task activation to subclusters and task activation without smoothing. Axial slices (left hemisphere on the left side) of the task activity (A, C and D) or the binary subcluster masks created with the watershed procedure (B) visualized using MRIcroGL. The activity for each contrast is thresholded at *p*_FWE_ < .05 and a cluster extent threshold based on *p* < .001 which corresponds to a z-value of 3.1. The render on the right shows a transparent overview of the activity in both hemispheres and the location of the axial slices. Activity or masks in blue are based on negative contrast maps and activity or masks in red are based on positive contrast maps. Panel A shows the task activation in the original analysis. Panel B shows the cluster segmentations after the watershed procedure. Panel C shows the task activation based on non-smoothed data. Panel D shows the task activation based on non-smoothed data in a low motion subgroup.

Whole brain analyses showed wide-spread task activation (Fig. 6). We found more activation during faces versus houses in the bilateral middle temporal gyrus, bilateral amygdala, left supramarginal gyrus, bilateral precuneus, and left precentral gyrus. Higher activity in houses versus faces was found in the bilateral fusiform gyrus and right superior occipital gyrus. See Table S3 for an overview of activation clusters. Higher activity in happy faces versus neutral faces was found in the bilateral occipital pole, right inferior occipital gyrus, left posterior orbital gyrus, left amygdala, left anterior insula, left middle cingulate gyrus, left middle frontal gyrus, and left superior frontal gyrus (Table S4). Higher activity in fearful faces versus neutral faces was found in the bilateral occipital fusiform gyrus, left inferior occipital gyrus, bilateral entorhinal area, right temporal pole, and bilateral thalamus (Table S5). Happy faces elicited more activation than fearful faces in the bilateral occipital pole, right inferior occipital gyrus, left anterior insula, left putamen, and left middle frontal gyrus (Table S6).

Inspired by Miller at al., 2016, we additionally visualized overlap in first-level activation patterns, i.e. the percentage of children passing simple voxel-wise activation thresholding (t > 1.96) for each contrast. Despite the widespread and strong activation patterns for all contrasts, only the activation in the bilateral fusiform gyrus extending to the superior occipital gyrus was robust and this cluster was significantly activated in over 50 % of the participants for the faces > houses (negative) contrast (Supplementary Figure S5). This suggests that this contrast elicits the most robust brain activation across individuals in our study.

Next, the large clusters were split up in subclusters based on the local peaks of the whole brain activation using a watershed procedure (Fig. 6). We ended up with 34 subclusters for faces > houses; 10 subclusters for houses > faces; 49 subclusters for happy faces > neutral faces; 15 subclusters for fearful faces > neutral faces; 50 subclusters for happy faces > fearful faces. These subclusters were then used for subsequent analyses.

#### 3.3.3 The effects of age and sex

**Fig. 7:**
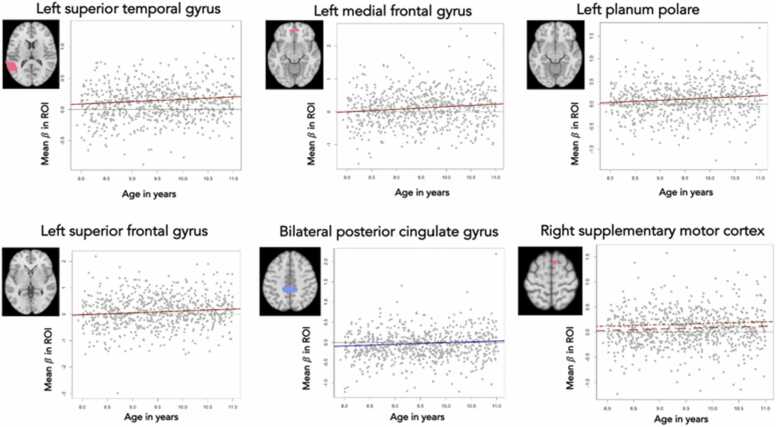
Age and sex effects on activity during faces versus houses. An axial slice shows the location of the subcluster (left hemisphere on the left side). In the plot individual *β*-weights from the second-level analysis, averaged over the ROI, are plotted against age in years. The solid red lines indicate that the contrast between faces > houses (positive) increases with age as group-average higher activity in faces than houses. The solid blue line indicates a large contrast in younger children with as group-average higher activity in the faces > houses (negative) contrast. The rightest plot in the bottom row shows the significant effect of sex with a larger contrast for faces > houses (positive) in girls and no age effect in this subcluster. The dot-dash line is based on the girls only and the dashed line is based on the boys only.

We did not find age or sex effects on activation patterns in the contrasts happy versus neutral (positive), fearful versus neutral (positive) and happy versus fearful (negative) (Supplementary tables S7 to S9). We did find a positive correlation between age and brain activity in the faces > houses contrast (i.e., larger contrast in older children; Fig. 7, Supplementary table S10). This effect was significant for 4 subclusters extracted from faces > houses (positive) in the left superior temporal gyrus, *t*(749) = 3.251, *p*_*FDR*_ = .0193, *p*_*uncorr*_ = .0012, *β* = 0.038 (SE = 0.012), the left medial frontal gyrus, *t*(749) = 3.415, *p*_*FDR*_ = .0193, *p*_*uncorr*_ = .0007, *β* = 0.078 (SE = 0.023), the left planum polare, *t*(749) = 3.225, *p*_*FDR*_ = .0193, *p*_*uncorr*_ = .0013, *β* = 0.051 (SE = 0.016), and the left superior frontal gyrus (medial segment), *t*(749) = 2.823, *p*_*FDR*_ = .0494, *p*_*uncorr*_ = .0049, *β* = 0.072 (SE = 0.026). Furthermore, we also found a positive correlation between age and brain activity in 1 subcluster extracted from faces > houses (negative) indicating less deactivation (i.e., higher activation) in older children for faces compared to houses in the bilateral posterior cingulate gyrus in older children, *t*(749) = 2.777, *p*_*FDR*_ = 0.0494, *p*_*uncorr*_ = .0056, *β* = 0.041 (SE = 0.015). Additionally, we found a larger contrast for faces > houses (positive) for girls in the right supplementary motor cortex, *t*(749) = 3.309, *p*_*FDR*_ = .0431, *p*_*uncorr*_ = .0010, *β* = 0.088 (SE = 0.026). No age effect was found in this subcluster.

#### 3.3.4 The relation with social competence

None of the four subscales of social competence were significant predictors of brain activity in any of the contrasts in linear models corrected for age and sex (Supplementary tables S11-S13).

### 3.4 The behavioral labeling and the neural processing of emotional faces

The ability to accurately label happy faces and the response time when correctly labeling happy faces did not significantly relate to brain activity in the happy versus neutral faces contrast (Supplementary table S14). Similarly, we did not find evidence that emotion labeling skills for fearful faces was related to brain activity during the processing of fearful versus neutral faces (Supplementary table S15). As a post-hoc analysis, we wondered if the most robust contrast (faces versus houses), would relate to response time during correct trials and accuracy in the emotion labeling task (independent of emotional valence). Again, we did not find significant associations between brain activity during faces versus houses and emotion labeling skills at a behavioral level (Supplementary table S16).

### 3.5 Post-hoc analyses

ROI-based task activation results with and without smoothing can be found in Table 2. For the contrast faces > houses (negative), we found an FDR-adjusted significant task effect in the bilateral fusiform gyrus, the bilateral occipital gyrus (inferior, middle and superior part), the bilaterial superior parietal gyrus and the right inferior temporal gyrus. Additionally, in the non-smoothed but not in the smoothed task activation data the bilateral inferior parietal gyrus was significantly activated in the contrast faces > houses (negative). For the contrast happy > neutral (positive), we found a significant task effect in the bilateral fusiform gyrus, the bilateral amygdala, the bilateral anterior cingulate gyrus, the bilateral middle occipital gyrus, the bilateral precuneus, the bilateral insula, the bilateral putamen, the bilateral supramarginal gyrus and the bilateral middle temporal gyrus. These regions thus showed higher activation in response to happy faces than in response to neutral faces. In the smoothed but not in the non-smoothed task activation data the bilateral medial orbital frontal gyrus was significantly activated in the contrast happy > neutral (positive). For the contrast fearful > neutral (positive), we found a significant task effect in the bilateral fusiform gyrus, the bilateral amygdala and the bilateral inferior occipital gyrus. These regions thus showed higher activation in response to fearful faces than in response to neutral faces. For the contrast fearful > happy (negative), we found a significant task effect in the bilateral anterior cingulate. These regions thus showed higher activation in response to happy faces than in response to fearful faces. No other differences were found between smoothed and non-smoothed data in the ROI analyses. Adding a frame-to-frame criterium for motion correction reduced the motion contamination in the data (Figure S6). The ROI-based task effects for the low motion subgroup with and without smoothing resembled the task effects in the original group with and without smoothing. Fig. 6 shows the impact of removing smoothing from the pipeline on whole brain task effects in the original group and the low motion subgroup. Whole brain task activation was widespread and scattered in many very small clusters in the non-smoothed data.

Subsequent analyses showed no evidence for effects of age, sex, emotion labeling accuracy, emotion labeling response time and social competence subscales on task activation in ROIs (all *p*_*FDR*_ > 0.05) in smoothed and non-smoothed data. Statistics corresponding to the subsequent non-significant ROI-analyses with and without smoothing can be found in the supplementary tables S17-S26. Analyses in the low motion subgroup also yielded no significant results (Table S27 to Table S36).

## 4 Discussion

In this study, we tested whether social competence, behavioral emotion labeling and neural processing of emotional faces were related in pre-adolescence and if inter-individual variation in these measures could be explained by age and sex. To this end we used data from 1054 children between 8- and 11-years-old participating in the YOUth cohort study. We found effects of age and sex on social competence, behavioral emotion labeling and neural face versus house processing, but no evidence for effects on neural differential processing of emotional expressions, nor for interrelations between social competence, behavioral emotion labeling and neural processing of emotional faces.

### 4.1 Social competence

We used four subscales to assess social competence (Junge et al., 2020). From the Interpersonal Reactivity Index (Davis, 1983) we used the subscales perspective taking (IRI-pt) and empathic concern (IRI-ec), and from the Strengths and Difficulties Questionnaire (Goodman, 1997, Goodman, 2001) we used the subscales peer problems (SDQ-pp) and prosocial behavior (SDQ-ps). The subscales were distributed as can be expected in a population-based study. Social competence was higher for girls than boys, consistent with literature (Maurice-Stam et al., 2018, Muris et al., 2003, Overgaauw et al., 2017). Furthermore, IRI-pt and SDQ-ps increased with age. Previous work shows age group differences between early adolescence and late adolescence for the IRI-pt but not the IRI-ec (Hawk et al., 2013). This may suggest that perspective taking (grouped under cognitive empathy) may have a protracted developmental trajectory compared to empathic concern (grouped under affective empathy). Using the SDQ, more prosocial behavior in older children (9–11 years) compared to younger children (7–8 years) have been reported before as well (Marzocchi et al., 2002, Marzocchi et al., 2004).

### 4.2 Behavioral emotion labeling

We assessed emotion labeling using the Penn CNB, a neurocognitive test battery with good validity and reliability (Swagerman et al., 2016). Accuracy reached a ceiling effect in pre-adolescence, especially for happy faces, neutral and fearful faces, leaving less room for inter-individual variation. Still, we show that in pre-adolescence there is an advantage in emotion labeling accuracy and response time for older children compared to younger children. Furthermore, girls were more accurate than boys in labeling happy, sad, and fearful expressions, and faster than boys in correct trials for happy and angry expressions. These associations are consistent with previous work using the Penn ER task in a population with a wider age range 8–21 (Gur et al., 2012). Furthermore, our findings are in line with age and sex effects found for emotion labeling accuracy, reaction time was not included, in 8- to 12-year-old children using the Radboud Faces Database (Verpaalen et al., 2019). It remains unclear if the age- and sex-effects on emotion labeling speed are domain-specific or reflect improvements in general cognitive ability or processing speed (Swagerman et al., 2016).

We did not find an association between emotion labeling accuracy or speed and any of the social competence subscales in models corrected for age and sex, contradicting previous studies. Previous work showed negative associations between emotion labeling accuracy and the SDQ-pp or total problem scores and positive associations between emotion labeling accuracy and SDQ-ps in young children (Burley et al., 2022), children with attention deficit hyperactivity disorder (Staff et al., 2022), children with neurodevelopmental disorders (Löytömäki et al., 2022), children with disruptive behavior (Hunnikin et al., 2020) and adopted children (Paine et al., 2023). For the IRI subscales, previous studies are less consistent. In a study on healthy adults, IRI-ec was negatively associated with accuracy in the Penn ER task (Beals et al., 2022), while in two other studies on adults IRI-ec was positively associated with emotion labeling accuracy (Israelashvili et al., 2020). Taken together, previous studies show that in young children, vulnerable populations and atypical developing children emotion labeling skills may be predictive of social competence. Here, we find no support that emotion labeling skills predict social competence which may be partly explained by the little variation in SDQ scores and by the ceiling effect for emotion labeling accuracy (see also Section 4.5 for a more extensive discussion).

### 4.3 Neural facial emotion processing

The fMRI task resulted in wide-spread activation for the faces > houses (positive and negative) contrast, the happy faces > neutral faces (positive) contrast, the fearful faces > neutral faces (positive) contrast and the fearful faces > happy faces (negative) contrast. Emotion faces elicited more activity than neutral faces and happy faces elicited more activity than fearful faces. When comparing our results to results in meta-analyses (Fusar-Poli et al., 2009, Xu et al., 2021, Liu et al., 2021), the activation in the current study is much more widespread. There are four possible explanations for this widespread activity. One, our sample size of 752 participants with fMRI data is much larger than the individual studies included in the meta-analysis. Large sample sizes can result in large clusters spanning multiple regions (Woo et al., 2014), something researchers can (partly) control for with stringent statistical thresholding. Two, the meta-analyses are mostly based on adults, and it is suggested that in childhood brain activity is more diffuse while maturation results in more focal activation patterns (Durston et al., 2006), even though this interpretation has also been criticized (Brown et al., 2006, Poldrack, 2010). Three, we found large inter-individual variation in the first-level contrast maps which may result in widespread second-level activation patterns. Four, for large developmental cohort studies, a smoothing kernel of 8 mm may result in too much smoothing (Gardumi et al., 2016, Jo et al., 2007; Sacchet and Knutson, 2013). We found minor difference when comparing results of smoothed and non-smoothed data in the ROI-analyses (Table 2). The whole brain task effects without smoothing still show widespread brain activity patterns although the large clusters are now scattered in numerous small subclusters (Fig. 6).

Activation patterns in the ROI-based and whole brain analyses partly overlapped with meta-analyses (Fusar-Poli et al., 2009, Xu et al., 2021, Liu et al., 2021). Importantly, based on literature (Fusar-Poli et al., 2009, Passarotti et al., 2003, Miller et al., 2016), we expected activity in the fusiform gyrus to be higher in faces compared to houses, but in our study fusiform gyrus activity was higher in response to houses as compared to faces. The different directionality in the fusiform gyrus may be explained by the inclusion of different age categories in our study compared to previous studies. Older participants showed greater neural response when processing emotional faces than younger participants in a meta-analysis (Fusar-Poli et al., 2009). Furthermore, the images of houses and faces may have differed on lower-level visual properties unrelated to the content (e.g. spatial frequency and variation in color). Therefore, some stimuli may have attracted more attention than others. Visual properties of the stimuli were not controlled for to keep stimuli more naturalistic. Alternatively, the use of different task stimuli could explain the different results: pictures of faces were contrasted to pictures of houses in the current study, and pictures of faces were contrasted to a fixation of a crosshair on the screen in Fusar-Poli et al. (2009).

Despite previous research showing that neutral faces can be ambiguous (Zhang et al., 2019), in our cohort the accuracy of labeling neutral faces was very high (Figure S3). Therefore, we believe that neutral faces were not necessarily a bad control in our study. Still, as the current design lacks in-scanner behavioral data, we are not able to differentiate between difficulties in distinguishing between emotions or differential activation related to the emotional valence of the stimulus. The passive-watching design of our study has more disadvantages. Because children were not actively labeling emotions, the attention and task engagement may have been lower. Still, in between stimuli blocks, children were instructed to press a button in response to a red circle and less than 1 % of the children did not respond to the red button stimuli in any of the trials and task compliance was good on average (Figure S4).

Another limitation of the current study is how motion was addressed. While the signal drop threshold removed extreme and repetitive motion artefacts, some level of motion contamination was still present in the data (Figure S6). To address this, we added a frame-to-frame metric for exclusion as a post-hoc analysis. We created a low motion subgroup by retaining children with at least 70 % low motion frames. After reviewing the literature, we defined low motion frames using a threshold of < 0.3 mm (Smith et al., 2022). A more stringent threshold may significantly impact our participant characteristics, as studies suggest that motion artefacts may be more pronounced in younger children, boys and children with ADHD or other diagnoses, though these factors are not consistently linked to motion across all studies (Thomson et al., 2024, Frew et al., 2022, Dosenbach et al., 2017). Results in our low motion subgroup were comparable to the results found in the larger original group. Although not incorporated in the current study, censoring high-motion data points in GLM designs is a way to reduce motion contamination in fMRI studies even more (Siegel et al., 2014).

To analyze effects of age, sex, social competence and emotion labeling on neural activity, we segmented the large clusters in subclusters with the aim of detecting the true signal seeds. Contrary to our expectations, no effects of age and sex were found for emotional versus neutral faces. There are no previous studies of this magnitude in 8-, 9- and 10-year-olds. Potentially, our brain-wide approach prevented us to pick up subtle effects (Marek et al., 2022), especially as fMRI data in general and for this task are only moderately reliable due to the state-dependent nature of brain function and other sources of variations such as noise (Buimer et al., 2020). Another explanation could be that the task design (passive-watching) did not elicit sufficient region-specific brain activity. Still, activity in four subclusters, that were more active during faces compared to houses, was positively associated with age with an increased contrast in older children in the left superior temporal gyrus, the left medial frontal gyrus, the left planum polare, and the medial segment of the left superior frontal gyrus. The activity in one subcluster that was more active during houses compared to faces was positively associated with age with a decreased contrast in older children in the bilateral posterior cingulate gyrus. Sex effects were found in one subcluster faces > houses (positive) with an increased contrast for girls in the right supplementary motor cortex. We did not find an association between neural processing and any of the social competence subscales. Previous studies did find associations between neural processing and the social competence subscales IRI-ec and IRI-pt, although not always. In adolescents, the IRI-pt was associated with seed-based functional connectivity with a negative association for most regions (Tremblay et al., 2022). Within the default mode network connectivity was positively associated with IRI-ec and IRI-pt in adolescence (Winters et al., 2021). In adults, activity in the bilateral superior medial frontal cortex (a node within the DMN) was positively associated with the IRI-pt and negatively with the IRI-ec (Oliveira-Silva et al., 2018). A study in adults using a false-belief task found positive associations between the IRI-pt and medial prefrontal cortex activity (False-Belief > False-Photograph), but no effect for the IRI-ec (Dodell-Feder et al., 2014). In young adults, functional brain activity in response to familiar versus unfamiliar faces was not related to the empathic concern subscale of the Interpersonal Reactivity Index (IRI-ec) (Heckendorf et al., 2016). No associations between neural activity during prosocial choices for friends and the IRI-ec or the IRI-pt were found in a study during mid-adolescence (Schreuders et al., 2019). In post-hoc analyses we repeated all analyses using ROIs based on previous literature instead of whole brain activity and no significant associations were found. Overall, in one of the largest studies to date in children between 8 and 11 years of age we find no support that social competence and neural processing of emotional faces are related contradicting smaller studies with different designs (resting-state functional connectivity or task-based fMRI with different tasks).

### 4.4 Association between behavioral emotion labeling and neural facial emotion processing

Previous research validated the Penn task in children and adults (Gur et al., 2010, Gur et al., 2012). A version of the emotion task was modified for use in the fMRI scanner and elicited task-specific activation in individuals older than 16 years (Roalf et al., 2014). Still, in this study we did not find an association between behavioral emotion labeling skills and the neural processing of emotional faces. One possible explanation is that variation in performance on the Penn task does rely on more than emotional processing brain networks, as motor speed, processing speed and cognitive ability play a role as well (Swagerman et al., 2016). In the same way, the passive watching task elicited widespread activity and may not have been able to selectively target the facial emotion processing network. In the same way, the social competence subscales included in this study may tap on different aspects of social behavior that are unrelated to emotion labeling or neural facial emotion processing. Lastly, the ceiling effect for emotion labeling accuracy could be an explanation that no link was found between emotion labeling and neural facial emotion processing (see also Section 4.5 for a more extensive discussion).

### 4.5 Emotion differentiation and labeling as building blocks of social competence

The current results also do not support prior suggestions that emotion differentiation and labeling are important building blocks of social competence (e.g. Bayet and Nelson, 2019; Junge et al., 2020). However, to find a direct relation between two tasks, there needs to be variation between individuals in both tasks. Such variation is often absent in tasks that show robust findings on a group level, referred to as the reliability paradox (Hedge et al., 2018). Indeed, the emotion labeling task in this cohort was selected because of their robustness on a group level (Onland-Moret et al., 2020) and shows little individual variation here. Most prior studies that show a strong relation between emotional labeling and social competence included groups that show more variation between individuals, because individuals either showed atypical development or were younger and strongly developing the skill. It could be hypothesized that the measurements of emotional labeling included in the current study were already matured well enough to not vary much between children, and therefore not directly relate to social competence anymore. Still, for neural emotion face processing the inter-individual variation was very high. Additionally, social competence relies on much more than emotion labeling or neural emotional face processing only. In each developmental period specific characteristics contributing to social competence are strengthened (Junge et al., 2020). Therefore, the link between emotion labeling and social competence may be stronger in younger children, while in pre-adolescence individual differences in social competence may be better predicted by complex cognitive processes such as attributing a mental state to someone; understanding the social context; determining what would be appropriate behavior under the circumstances (Hoemann et al., 2019). Recent studies experiment with more naturalistic or dynamic emotional stimuli (for example, Camacho et al., 2023).

### 4.6 Conclusion

We tested for interrelations between three predictors of social behavior in daily life: social competence, emotion labeling and neural processing of emotional faces. In a developmental cohort of pre-adolescents, we show an advantage for girls and older children for social competence and facial emotion labeling, but no support for a relation between the two factors. Furthermore, we show strong and widespread brain activity in response to faces (happy faces > fearful faces > neutral faces) and houses, but no association between the task contrasts and social competence or behavioral emotion labeling. To conclude, we find age- and sex-related variation in emotion labeling skills and social competence in pre-adolescence. However, in a population cohort we did not find support for associations between neural activity in response to faces, behavioral emotion labeling and social competence.

## CRediT authorship contribution statement

**Mathijs Raemaekers:** Writing – review & editing, Software, Methodology, Conceptualization. **Rachel M. Brouwer:** Writing – review & editing, Writing – original draft, Validation, Supervision, Software, Resources, Methodology, Investigation, Formal analysis, Conceptualization. **Hilleke E. Hulshoff Pol:** Writing – review & editing, Writing – original draft, Visualization, Validation, Supervision, Software, Resources, Project administration, Methodology, Investigation, Funding acquisition, Formal analysis, Conceptualization. **Elizabeth E.L. Buimer:** Writing – review & editing, Writing – original draft, Visualization, Validation, Software, Project administration, Methodology, Investigation, Formal analysis, Data curation, Conceptualization. **Pascal Pas:** Writing – review & editing, Writing – original draft, Visualization, Validation, Software, Resources, Project administration, Methodology, Investigation, Formal analysis, Data curation, Conceptualization. **Carlijn van den Boomen:** Writing – review & editing, Conceptualization.

## Funding

YOUth was funded through the Gravitation program of the Dutch Ministry of Education, Culture, and Science and Netherlands Organisation for Scientific Research (NWO grant number 024.001.003). YOUth is part of (and partly funded by) the research theme Dynamics of YOUth of Utrecht University and of the UMC Utrecht Brain Center. The Consortium on Individual Development (CID) is funded through the Gravitation program of the Dutch Ministry of Education, Culture, and Science and the Netherlands Organization for Scientific Research (NWO grant number 024.001.003).

## Declaration of Competing Interest

The authors declare that they have no known competing financial interests or personal relationships that could have appeared to influence the work reported in this paper.

## Data Availability

Data will be made available on request.
