# Supplementary material for: Age- and sex-related differences in social competence and emotion labeling in pre-adolescence
Source: Dev Cogn Neurosci. 2024 Dec 24;71:101503. doi: 10.1016/j.dcn.2024.101503 (PMC11743816; doi:10.1016/j.dcn.2024.101503)
Supplement: Supplementary file 1 — Supplementary material [file mmc1.docx]

### Supplementary figures

*Tables can be found in supplementary excel file.*

**
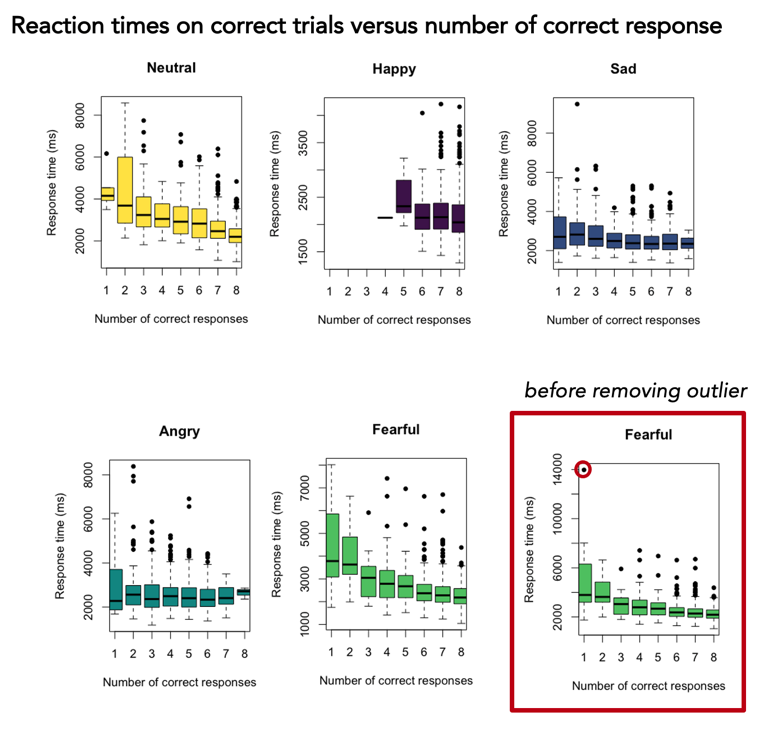
**

**Figure S1. Reaction for correct trials versus number of correct trials.** We removed one participant from the final dataset because of the slow response time (14 seconds, i.e., 13 standard deviations from the mean) based on only one correct trial.

**
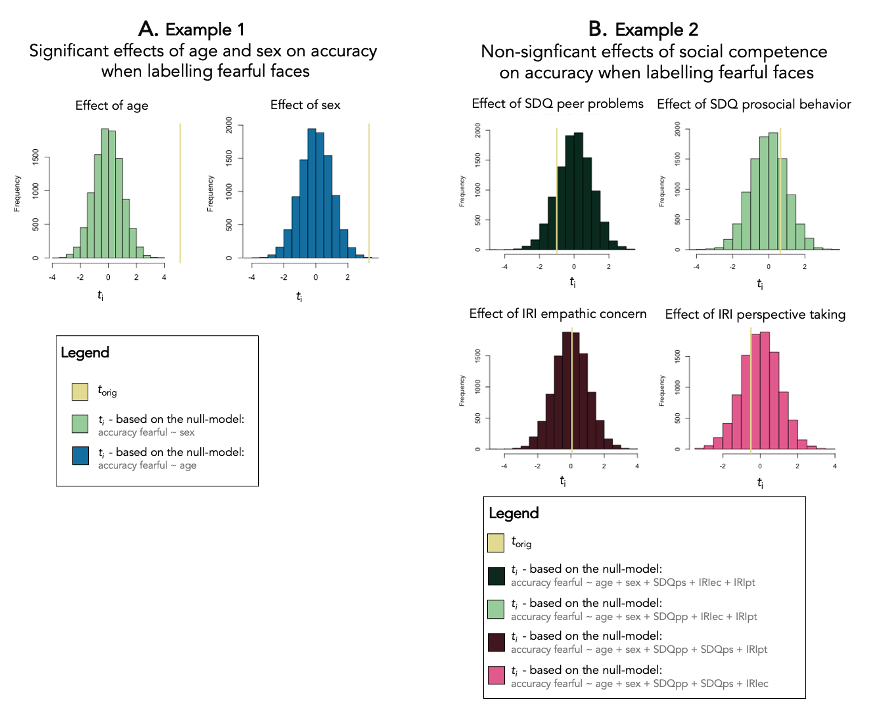
Figure S2. Examples of residual-based permutations.** A. Results of residual-based permutations for the effects of age and sex on accuracy when labeling fearful faces. B. Results of residual-based permutations for the effects of social competence subscales on accuracy when labeling fearful faces.


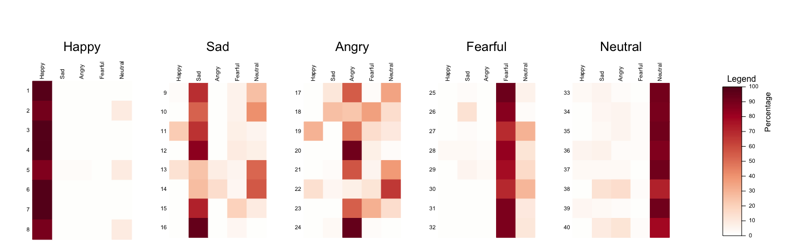


**Figure S3. Accuracy for emotion labeling for each trial.** Each row is a specific actor expressing the emotion indicated above the graph. The columns in each graph show the responses by the children (from left to right: happy, sad, angry, fearful or neutral). The color indicates the proportion of children picking a certain response.


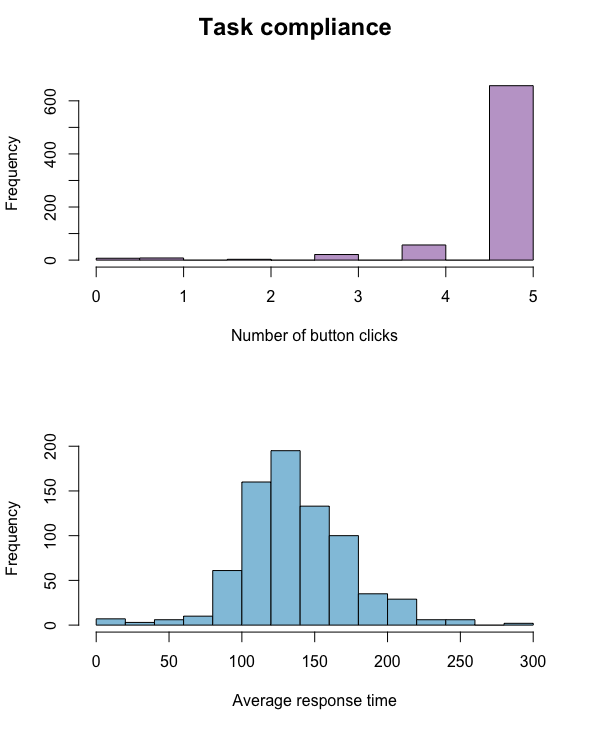


**Figure S4. Task compliance in the fMRI task.**


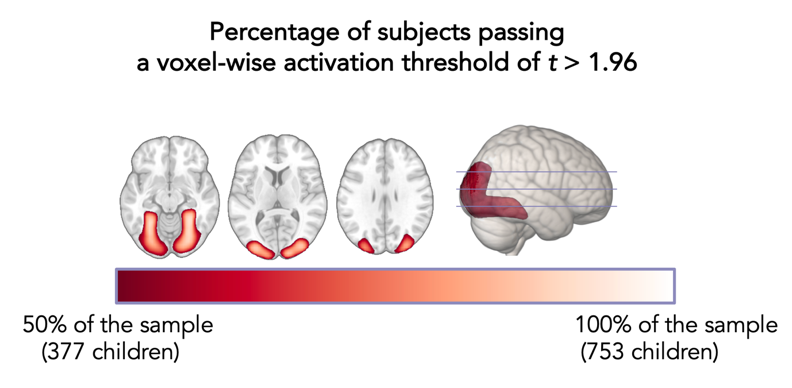


**Figure S5. Overlap in first-level activation patterns across individuals for faces > houses (negative).** The percentage of children passing simple voxel-wise activation thresholding (t > 1.96). Faces > houses (negative) was the only contrast where we found voxels that were significantly activated in at least half of the sample. This method was inspired by Miller et al., 2016 and visualized using MRIcroGL.

**
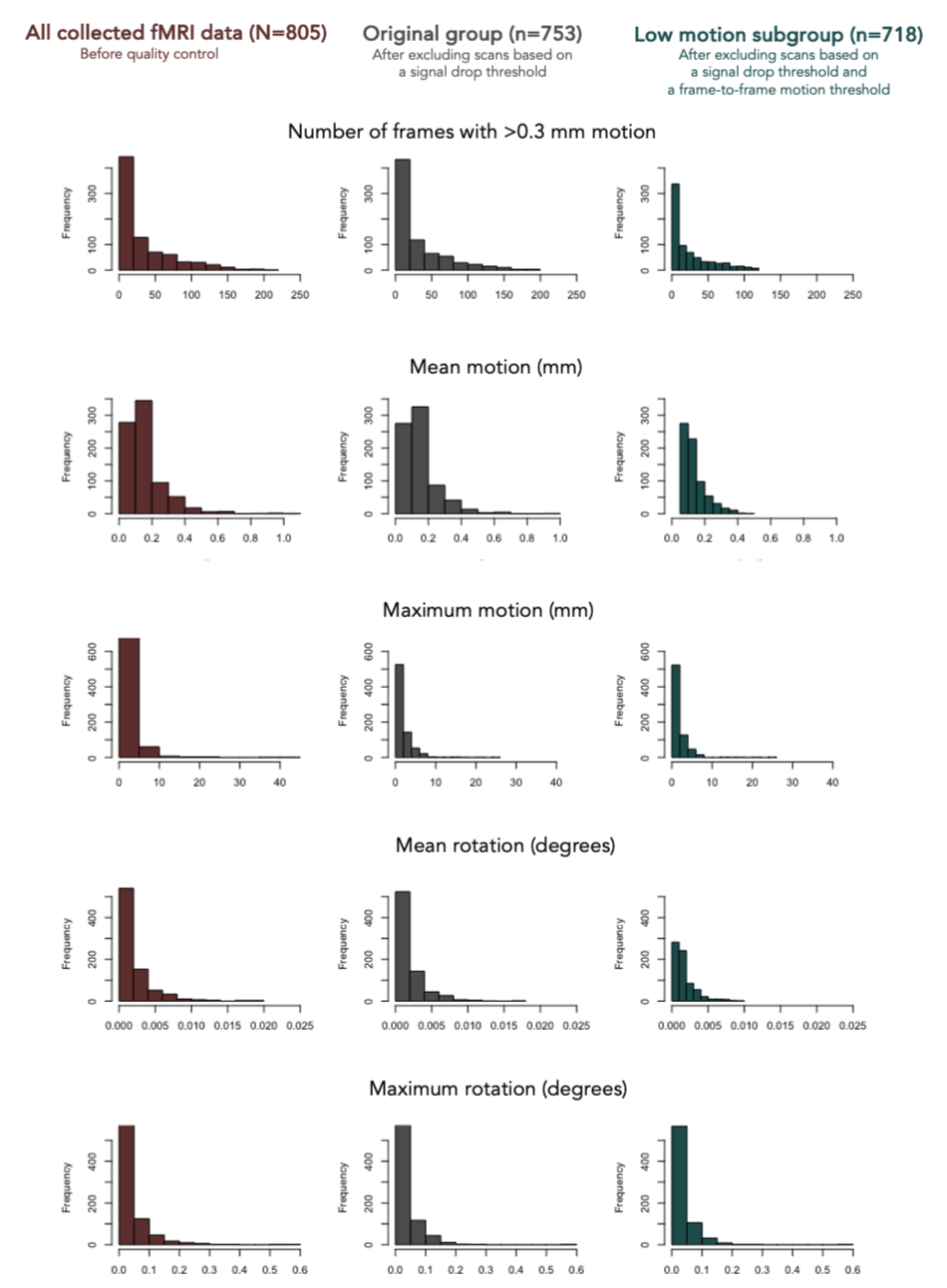
Figure S6. Motion artefacts and the effect of excluding data based on a signal drop threshold (original group) and a frame-to-frame motion threshold in addition to the signal drop threshold (low motion group).**

**References**

Miller, K. L., Alfaro-Almagro, F., Bangerter, N. K., Thomas, D. L., Yacoub, E., Xu, J., Bartsch, A. J., Jbabdi, S., Sotiropoulos, S. N., Andersson, J. L. R., Griffanti, L., Douaud, G., Okell, T. W., Weale, P., Dragonu, I., Garratt, S., Hudson, S., Collins, R., Jenkinson, M., … Smith, S. M. (2016). Multimodal population brain imaging in the UK Biobank prospective epidemiological study. Nature Neuroscience, 19(11), 1523–1536. <https://doi.org/10.1038/nn.4393>
